# Iron Supplements Containing *Lactobacillus plantarum* 299v Increase Ferric Iron and Up-regulate the Ferric Reductase DCYTB in Human Caco-2/HT29 MTX Co-Cultures

**DOI:** 10.3390/nu10121949

**Published:** 2018-12-08

**Authors:** Ann-Sofie Sandberg, Gunilla Önning, Niklas Engström, Nathalie Scheers

**Affiliations:** 1 Department of Biology and Biological Engineering, Chalmers University of Technology, SE-41296 Gothenburg, Sweden; ann-sofie.sandberg@chalmers.se (A.-S.S.); niklas.engstrom@chalmers.se (N.E.); 2 Biomedical Nutrition, Pure and Applied Biochemistry, Center for Applied Life Sciences, Lund University, SE-22100 Lund, Sweden; gunilla.onning@probi.com; 3 Probi AB, SE-22370 Lund, Sweden

**Keywords:** *Lactobacillus plantarum* 299v, DSM9843, iron, DCYTB, MUC5AC, DMT1

## Abstract

Several human interventions have indicated that *Lactobacillus plantarum* 299v (*L. plantarum* 299v) increases intestinal iron absorption. The aim of the present study was to investigate possible effects of *L. plantarum* 299v on the mechanisms of iron absorption on the cellular level. We have previously shown that lactic fermentation of vegetables increased iron absorption in humans. It was revealed that the level of ferric iron [Fe (H_2_O)_5_]^2+^ was increased after fermentation. Therefore, we used voltammetry to measure the oxidation state of iron in simulated gastrointestinal digested oat and mango drinks and capsule meals containing *L. plantarum* 299v. We also exposed human intestinal co-cultures of enterocytes and goblet cells (Caco-2/HT29 MTX) to the supplements in order to study the effect on proteins possibly involved (MUC5AC, DCYTB, DMT1, and ferritin). We detected an increase in ferric iron in the digested meals and drinks containing *L. plantarum* 299v. In the intestinal cell model, we observed that the ferric reductase DCYTB increased in the presence of *L. plantarum* 299v, while the production of mucin (MUC5AC) decreased independently of *L. plantarum* 299v. In conclusion, the data suggest that the effect of *L. plantarum* 299v on iron metabolism is mediated through driving the Fe^3+^/DCYTB axis.

## 1. Introduction

### 1.1. Lactobacillus plantarum 299v and Iron Absorption

There are various forms of iron supplements available, used by, for example, pregnant women and young girls to treat or prevent iron deficiency. Iron supplements are often associated with adverse effects in the gastrointestinal tract and therefore we need new ways of supplementing iron that are better tolerated by the intestine. Probiotic bacteria, or live bacteria that are associated with positive health effects related to the gastrointesinal tract, could therefore be beneficial components in iron supplements in order to lessen or alleviate the potential side effects of iron salts. Probiotic effects are often strain-specific and may depend on the way the supplements are administered, for example, as a non-metabolically active supplement (lyophilized), as live (viable) bacteria added to a product, or as a fermented product. The effect of probiotic bacteria of the strain *L. plantarum* 299v on iron absorption in humans has been investigated in several instances with conflicting results, which may indicate that the way of administrating the bacteria determines the outcome. Examples that may illustrate this are two studies of slightly different experimental conditions from the same research group, which indicated in one of the studies that a lactic fermented (*L. plantarum* 299v) oat gruel increased iron absorption in healthy young women [1] and did not increase iron absorption when slightly or non-metabolically active bacteria was added to the heat-inactivated fermented oat gruel [2]. 

Since the addition of live bacteria to the heat-treated fermented product did not increase iron absorption, it seems that it is the crude *fermented* product and not the bacteria that is responsible for the beneficial effect on iron absorption. Four single-blind, sequential, placebo-controlled human interventions with *L. plantarum* 299v, conducted by another research group, gave inconsistent results, with three out of four successful studies indicating an improved iron absorption in the presence of the bacteria [3,4]. In two of the interventions, *L. plantarum* 299v were administered in a lyophilized encapsulated form and in the other two the bacteria were added live to oat and mango drinks. These studies did not support the older studies [1,2] in terms of the impact related to the administration. These four studies (and two more) were used for a health claim application to the European Food Safety Authority (EFSA) stating that *L. plantarum* 299v increases non-heme iron absorption in humans. However, the application was not approved and the EFSA concluded that there is insufficient scientific evidence to support such a claim [5]. One reason for this conclusion was that no plausible mechanism was presented in the application. This is where the present study comes in, in which we investigated the effects of the same encapsulated supplement and fruit drinks (oat or mango) as in the four studies on iron absorption, but this time at the mechanistic level in a human intestinal co-cultured cell model (Caco-2/HT29 MTX).

### 1.2. Intestinal Uptake Routes for Iron

Iron absorption has traditionally been described to mainly involve two routes, either as active uptake of heme-bound iron (through the folate transporter HCP1) or the transport of inorganic iron into the cell through DMT1 (DMT1A and DMT1A-IRE). Today, we also know that iron may be absorbed by the endocytosis of large iron complexes, which is thought to dissolve lysosomatically, and then enter the cytosol through DMT1 [6]. It is not clear if the transport from the lysosome to the cytosol involves the same isoform of DMT1 as the transcellular transport across the lumen-intestinal interface. The DMT1 isoforms DMT1B and DMT1B-IRE have been associated with cytosol-endosome transport of transferrin-imported iron from the basal cytosol [7]. DMT1 (NRAMP2/SLC11A2/DCT1) is a cation H^+^-coupled transporter that is mainly distributed along the ileum and at high density in the duodenum.

The human form (hDMT1) has been suggested to prefer ferrous iron (Fe^2+^) compared to other divalent cations [8]; in one case, hDMT1 was shown to have higher affinity for Cd^2+^ than Fe^2+^ [9]. Iron in the gastrointestinal lumen is mostly present in oxidized form (Fe^3+^) and must therefore be reduced before it can be transported by DMT1. Ferric iron that has not been reduced by luminal molecules, such as ascorbic acid, can then be reduced by a membrane-spanning reductase, DCYTB, in the intestinal epithelium [10]. Also, DCYTB and DMT1 have been shown to co-localize at the luminal border (in rat). Once ferrous iron is transported into the cells, it is rapidly oxidized to the ferric form again and either will be incorporated into the cellular LIP (labile iron pool) or transported by means of chaperones targeting cytosolic ferritin, in which iron is stored for later use. Cytosolic ferritin levels have been shown to be proportional to cellular iron uptake and are therefore often used as a proxy for iron uptake.

### 1.3. The Present Study

The study design aimed at investigating if iron uptake can be increased in the presence of *L. plantarum* 299v and to study specific questions related to the mechanism of the effect on iron absorption. In previous studies, we observed that the oxidation state of iron favours that of Fe^3+^ to a significant extent after lactic fermentation of vegetables [11] and carrot juice [12], and therefore one of the questions here was if the lyophilized bacteria or the live viable bacteria added to the oat and mango drinks also had the capacity to increase iron in the oxidized form, ferric iron. After confirming that both formulations increased the level of ferric iron, the next aim was to investigate the consequence of the increased load of ferric iron on iron transport, and therefore the effect on DCYTB and DMT1 was studied. Intestinal mucus has been shown to have high affinity for ferric iron and has been proposed to serve as a reservoir for iron [13]. Additionally, the expression of intestinal mucins has been observed to increase in the presence of probiotic bacteria [14], so therefore another question was how the ferric load and the presence of *L. plantarum* 299v affect the production of mucus. We chose to investigate these questions in a previously described human co-culture model that combines intestinal cells with mucus-producing goblet cells, at an optimized ratio, to be used for iron bioavailability studies [14].

## 2. Materials and Methods 

### 2.1. Bacterial Strain and Capsule Formulation 

The bacterial strain investigated in the study was *Lactobacillus plantarum* 299v (DSM 9843). The active capsule contained lyophilized *L. plantarum* 299v at 10^10^ CFU, Fe fumarate (4.2 mg), ascorbate (12 mg), and folic acid (30 μg). The control capsule contained the same constituents but no bacteria. The capsule itself was composed of hydroxypropylmethyl cellulose and was designed to dissolve in the stomach after 20–25 min.

### 2.2. Composition of Meals, Oat and Mango Drinks

The capsule meals, oat and mango drinks had the same composition as previously described [3,4]. The meals to go with the capsules consisted of two bread rolls (for recipe, see [3]), a Flora sandwich spread (15 g; Flora, Unilever, London, UK), Önos orange jam (20 g; Önos; Orkla Foods, Malmö, Sweden), water (200 mL), and one capsule (with or without *L. plantarum* 299v). A batch of meals (based on 12 buns, without capsules) were prepared and partly homogenized in a chopper (Kenwood, Akaho, Japan). The slurries were weighed and immediately frozen in Falcon tubes (56.6–61.0 g in each tube). The total weight of the meal batch was 2091.2 g.

Oat drinks and mango drinks with and without *L. plantarum* 299v were prepared and all drinks were supplemented with iron. Ascorbic acid content was unmodified (Table 1). The oat drink with *L. plantarum* 299v was the same as used in the trial by Hoppe et al. [4] in which the bacteria were added as a fermented oat gruel. The mango drink with *L. plantarum* 299v was similar, but the bacteria was added as a ferment without oats.

### 2.3. Simulated Gastrointestinal Digestion of Capsule Meals

Active capsule (with *L. plantarum* 299v) and capsule control (without *L. plantarum* 299v) were added to two different beakers (2 dm^3^) that contained 348.5 g meal slurry each (equivalent to one meal). Next, α-amylase solution (350 mL; 75 U per mL in 0.9% NaCl; 37 °C) was added to the beaker and the digest was incubated for 2 min (150 rpm; 37 °C). The pH at this stage was 5.12. The capsules were not visible at the surface at any time during the incubation. Pepsin solution was thereafter added to the digest (350 mL; 2000 U per mL in 0.1 M HCl; 37 °C) and the pH became 1.69 in the Lp299v capsule digest and 1.65 in the capsule control digest. The incubation lasted for 30 min (150 rpm; 37 °C). Proceeding with the digestion, pH was stepwise increased with NaHCO_3_ (1M) to 6.67 in the capsule meal and 6.70 in the capsule control meal. The digest was thereafter left for incubation for 1 h (150 rpm; 37 °C).

### 2.4. Simulated Gastrointestinal Digestion of Oat and Mango Drinks

The oat and mango drinks (1 mL) were mixed with water (5 mL; Ultrapure water type 1, and α-amylase solution (5 mL; 75 U per mL in 0.9% NaCl; 37 °C). The drinks were incubated for 2 min (150 rpm; 37 °C) before pepsin solution was added (5 mL; 2000 U per mL in 0.1 M HCl; 37 °C). The drinks were further incubated for 30 min (150 rpm; 37 °C) and then the pH was raised to 6.8 by adding NaHCO_3_ (1M). The digest was thereafter left for incubation for 1 h (150 rpm; 37 °C).

### 2.5. Differential Pulse Anodic Stripping Voltammetry (DPASV) Measurements

Iron speciation (Fe^2+^/Fe^3+^) of the digested fruit drinks and capsule meals were analysed with Differential Pulse Anodic Stripping Voltammetry (DPASV) using the Computrace 797 (Metrohm Nordic AB, Stockholm, Sweden) with a platinum rotating disc electrode (Pt-RDE), a platinum auxiliary electrode, and an Ag/AgCl/KCl reference electrode, as previously described [12]. All measurements were done at pH 3.9–4.0 in a water solution containing NaClO_4_ (0.1 M) as the conductive medium. The pH was chosen according to Allen and Flemström, who estimated the average pH in the upper duodenal lumen to be 3.99 one hour after food intake (in humans). The pH was set by addition of HCl (1 M) and an external pH-meter was mounted to the reaction vessel to monitor the stability of the pH. In addition, a thermostat jacket was connected to keep the temperature at 37 °C. Digested samples were added to the vessel (700 μL; the total volume was 15 mL) for measurements.

### 2.6. Experiments in the Caco-2/HT29 Co-Culture Cell Model

Human intestinal cells (Caco-2; HTB37; ATCC, Manassas, VA, USA) and mucus-producing goblet cells (HT29-MTX-E12; ATCC, VA, USA) were grown separately in flasks and the medium (MEM, 10% FBS; Gibco; Thermo Fisher Scientific; Waltham, MA, USA) was changed three times a week. Cells were passaged at approx. 80% confluence about once per week. The cells were seeded in 12-well plates in the ratio 75% Caco-2 cells (p.37–39)/25% HT29-MTX cells (p.52–54) according to the methods of Mahler et al. [15] and co-cultured for 13 days before the experiments. At day 13, the medium was changed into MEM 5% FBS. Twenty-four hours later, at day 14, the experiments were initiated. In the first trials, the cells were incubated with the content of the *L. plantarum* 299v and control capsules in triplicates for 5–60 min. After that, the cells were washed in PBS, new warm medium (MEM 5% FBS) was added, and then the cells were brought back to the incubator for another 24 h to allow them to respond in terms of changing their protein expression. At day 15, each set of cells from the five time points + controls (0 h) were lysed in RIPA buffer (Sigma-Aldrich, Schnelldorf, Germany) containing Pierce protease/phosphatase inhibitors (Thermo Fisher Scientific; MA, USA). Ferritin formation was measured with a DRG ferritin kit (EIA-4408; DRG, CA, USA) and DMT1, DCYTB, and MUC5A1C were all measured with specific ELISAs based on HRP cleavage of substrate, yielding either a fluorescent or coloured product (Amplex® Elisa development kit, Invitrogen, Paisley, UK or o-Phenylenediamine dihydrochloride, Sigma-Aldrich, Schnelldorf, Germany). DMT1, DCYTB, and MUC5A1C protein expression was normalised to cellular protein in each well (Pierce BCA assay; Thermo Fisher Scientific; MA, USA). In the second set of trials, the cells were incubated with digested oat and mango drinks ([Fe] = 16 µM) and capsule meals for 4 h ([Fe] = 29 µM). The cells were then brought back to the incubator for 20 more hours before lysis (24 h in total).

### 2.7. Statistics

All statistical analyses were done in Microsoft Excel 2011–2017. Significance tests were made using Student’s unpaired 2-tailed *t*-test and *p* values <0.05 were considered to be significant. All experiments were done in triplicates; data are means ± standard deviation (SD) from *n* = 2–4, where *n* is the number of trials.

## 3. Results

### 3.1. *L. Plantarum* 299v-Induced Increase in Ferric Iron in Simulated Gastrointestinal Digested Oat Drinks Was Associated with Elevated Expression of the Cellular Ferric Reductase DCYTB in the Caco-2/HT29 MTX Cell Model

**Figure 1:**
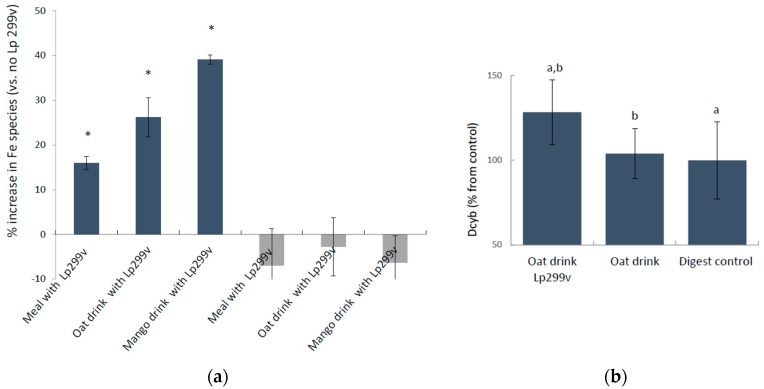
(**a**) Blue bars: percentage increase in ferric iron (Fe^3+^) and grey bars: insignificant changes (*p* < 0.05) in ferrous iron (Fe^2+^) in *L. plantarum* 299v supplemented meals, oat and mango drinks after simulated gastrointestinal digestion measured with differential pulse anodic stripping voltammetry (DPASV). Data are means ± SD, *n* = 3. An asterisk (*) indicates a significant difference from control (without *L. plantarum* 299v; *p* < 0.05). (**b**) Cellular level of the ferric reductase DCYTB was significantly increased (24%; *p* = 0.027) after a 4-h incubation with digested oat drinks containing *L. plantarum* 299v. DCYTB protein levels in cells (triplicate wells) were measured after 24 h from the first encounter. Data are means ± SD, *n* = 2. Letters a and b indicate significant differences (a: *p* = 0.032, b: *p* = 0.027).

The oxidation state of soluble iron (Fe^3+^/Fe^2+^) in the simulated gastrointestinal digested capsule-meals and drinks with and without *L. plantarum* 299v was compared using differential pulse anodic stripping voltammetry, which measures ferric and ferrous species simultaneously in the solution. The presence of *L. plantarum* 299v in the digested capsule meals and drinks significantly increased the level of ferric iron by 16% ± 1.49%, *p* = 0.017 (capsule meals), 26% ± 4.4%, *p* = 0.001 (oat drink), and 39% ± 1.0%, *p* = 0.019 (mango drink) (Figure 1a). There was no change in the levels of soluble ferrous iron, suggesting that the ferric iron is released from the matrix in the presence of the bacteria. The results also suggest that the actual fermentation (the level of metabolic activity) might increase ferric iron, since the smallest increase was represented by the lyophilized bacteria, and the greatest increase by the live bacteria in the oat and mango drinks. Oat drink digests were fed to the Caco-2/HT29 MTX cell model (4-h incubations) and the cells responded with an increase in the protein expression of the brush boarder ferric reductase, DCYTB, in the presence of *L. plantarum* 299v (24%, *p* = 0,027) (Figure 1b).

### 3.2. Time-Response Experiments of Undigested Capsule Contents in Caco-2/HT29 Cells

#### 3.2.1. The Reductase DCYTB and the Ferrous Iron Importer DMT1

**Figure 2:**
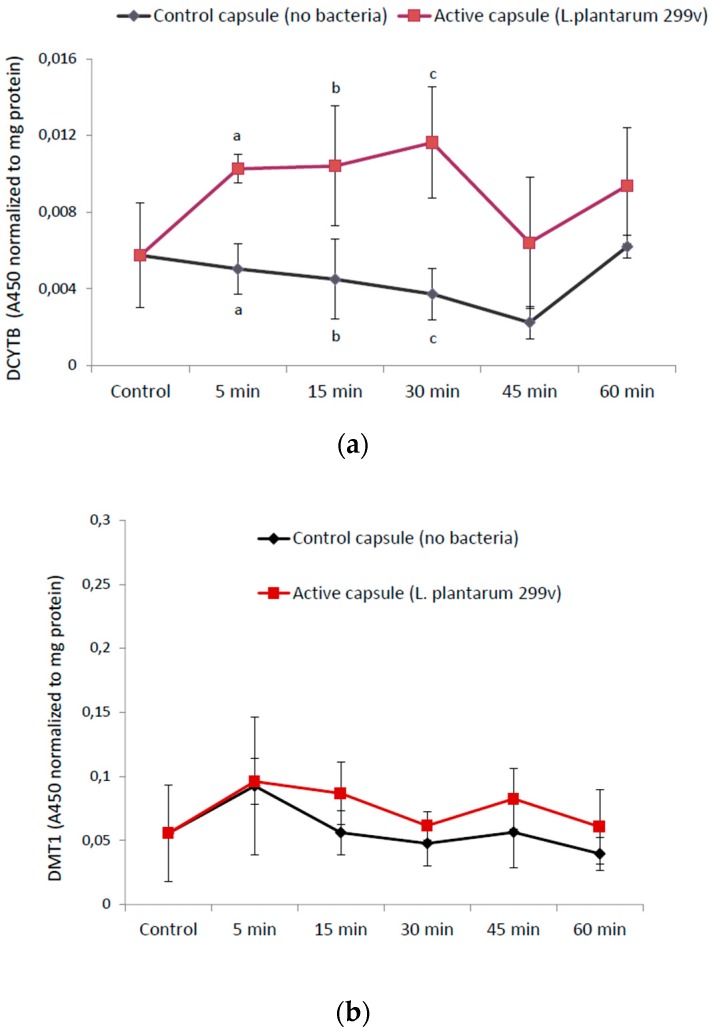
(**a**) Cellular level of the ferric reductase DCYTB was significantly increased in the presence of *L. plantarum* 299v (*p* = 0.0008 at 5 min, *p* = 0.03 at 15 min, *p* = 0.004 at *t* = 30 min) as indicated with letters a, b, and c. (**b**) Cellular expression of the iron importer DMT1 was not significantly increased in the time interval 5–60 min of incubation, as measured 24 h after the first encounter. Data are means ± SD, *n* = 3.

The investigation also showed that there is an immediate interaction between the lyophilized *L. plantarum* 299v in the supplement (containing iron, ascorbic acid, and folic acid) and the intestinal cells, which only required 5 min of incubation (Figure 2a). An increase in the capacity to reduce ferric iron (Fe^3+^) into ferrous iron (Fe^2+^) in association with increasing levels of ferric iron (Fe^3+^) in the presence of bacteria could be part of a potential positive effect of *L. plantarum* 299v on iron absorption. Accordingly, we investigated if an increase in ferric reductase activity would also affect the ferrous iron (Fe^2+^) importer DMT1. The data indicated that there was no significant effect of *L. plantarum* 299v on DMT1 expression (5–60 min), Figure 2b.

#### 3.2.2. Cellular Mucin (MUC5AC) Production

**Figure 3:**
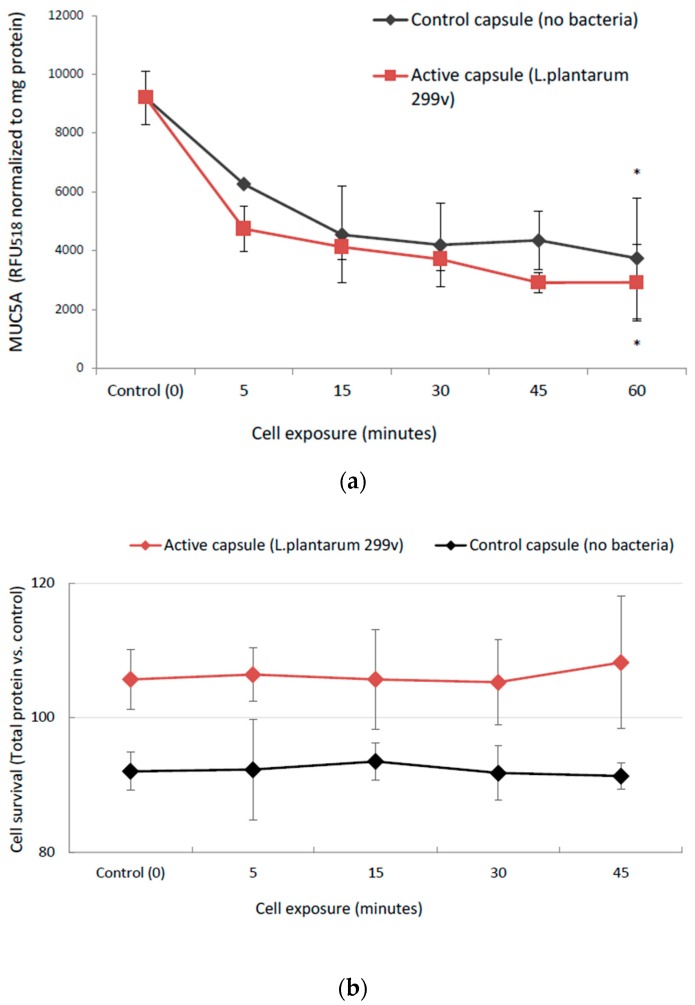
(**a**) Mucin production (MUC5AC) significantly decreased from baseline (control, no exposure to any capsules) in the presence of the capsules independent of *L. plantarum* 299v (from 0 to 60 min; *p* = 0.008 and *p* = 0.002 for control and active capsules, respectively). * indicates a significant difference from control; Data, shown as relative fluorescence units (RFU) normalized to total cell protein, are means ± SD, *n* = 3. (**b**) Total protein (proportional to cell number) shows that the decrease in mucus production is not caused by cell death.

We used MUC5AC as a marker for mucus production, since MUC5AC is readily expressed in the goblet cell line HT29-MTX-E12 [16]. Both the control capsule and the capsule containing *L. plantarum* 299v affected the cells to downscale their expression of MUC5AC, suggesting that this effect may be caused by the supplemental iron, ascorbic acid, or folic acid rather than the bacteria (Figure 3a). The decrease in mucus production was not caused by cell death (Figure 3b). The level of secreted mucin into the medium was fairly constant (data not shown) in all treated cells. The difference between the control cells with no capsule (*t* = 0) and cells with capsules was significant after 60 min of incubation (control capsule: *p* = 0.008 and *L. plantarum* 299v capsule: *p* = 0.002). There were also small significant differences between the control and *L. plantarum* 299v capsule at two time points in which the mucin production was lower in cells incubated with *L. plantarum* 299v (5 min: *p* = 0.05 and 45 min: *p* = 0.01)

### 3.3. Cellular Uptake of Iron (Ferritin Expression) in Response to L. Plantarum 299v

**Figure 4:**
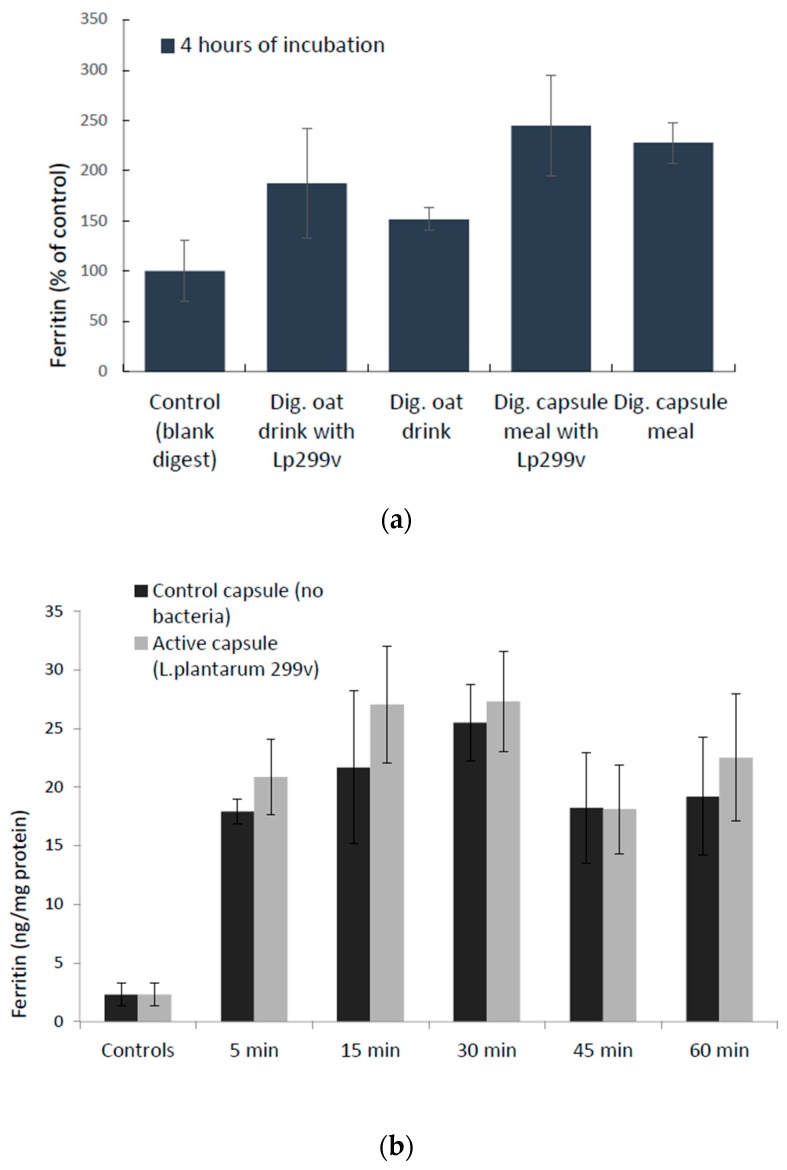
Iron uptake (ferritin expression) in Caco-2/HT29 MTX cells. (**a**) Data are means ± SD, *n* = 3 for digested (dig.) oat drink trials and *n* = 2 for capsule meal trials; each trial was done in triplicates. The differences between the study products and their controls (no bacteria) were not significant. (**b**) Cellular ferritin levels in Caco-2/HT29 MTX cells in response to the content of iron capsules, with and without *L. plantarum* 299v. Data are means ± SD, *n* = 2, in which each trial was done in triplicates. The differences between active capsules and control capsules (no bacteria) were not significant.

Caco-2/HT29 MTX cellular uptake of iron, estimated by measuring a proxy for iron uptake, ferritin, was not significantly increased due to the presence of *L. plantarum* 299v after a 4-h incubation with digested oat drinks (23%, *p* = 0.3) or a capsule meal (7%, *p* = 0.8). Nor did we observe a significant increase in ferritin expression in the presence of lyophilized *L. plantarum* 299v compared to capsule content with no bacteria in the time-response study (5–60 min), which supports the results from the 4-h incubation study (digested oat drinks and meals), in which the increase also was insignificant (Figure 4).

## 4. Discussion

The novel finding of this study that the intestinal border ferric reductase DCTYB increases in the presence of *L. plantarum* 299v indicates that *L. plantarum* 299v affects iron metabolism. However, it is not clear if this is a secondary effect of the increase in ferric iron, although it seems plausible since ferric iron is a substrate to DCYTB. In addition, comparing the graphs in Figure 1, it is evident that the increase in ferric iron in active oat drink (26%) is similar to the increase in cellular DCYTB (24%) in response to the same drink, which supports such a reasoning. However, it might not explain why the authors in the studies by Bering et al. (described in the introduction) [2] did not observe an increase in iron absorption after heat treating the fermented gruels following the addition of live or lyophilized bacteria. It would have been interesting to see data on the ferric/ferrous iron content before and after the heat treatment in order to evaluate if this process affected the oxidation state of iron.

One can also argue that the immediate effect of a 5-min incubation (+24-h delay until lysis) of *L. plantarum* 299v on DCYTB expression may imply that the effect on DCYTB does not require any extent of fermentation of an iron-containing matrix. Examining this statement further, a 5-min incubation, in addition to possible remaining bacterial cells after the washing step, which then had 24 h of further incubation, may just be sufficient to affect the state of iron in the serum-containing medium. Also, the cells were exposed to about 80% higher concentration of iron (29 µM) from the capsules compared to the drink digests (16 µM), which may explain the seemingly larger increase in DCYTB levels in response to the capsules. To conclude the reasoning, there is no obvious contradiction to the statement that the increased DCYTB expression may be a secondary effect of the increase in ferric iron.

Another question that appears is why the iron importer DMT1 did not increase accordingly. The simple answer may be that the initial increase in ferric iron is not likely to stimulate the expression of DMT1, since its substrate is ferrous iron and not ferric iron. One would expect that succeeding the reduction of ferric iron by DCYTB, the increase in ferrous iron would initially upregulate, and later downregulate, DMT1. The presence of ascorbic acid may also play a role in the effect [17]. In another study, in the same cell model (Caco-2/HT29 MTX), in which the effects of probiotic bacteria, including *L. plantarum* 299v, on intestinal barrier integrity with and without caprine milk carbohydrates, *L. plantarum* 299v was observed to reduce MUC5A expression [18]. However, in the present study, we observed a decrease in MUC5A expression in response to the capsules, *independent* of the presence of *L. plantarum* 299v.

Through the years, there have been several suggested reasons for the enhancing effect of fermented foods on iron absorption, such as the production of lactic acid and decrease in pH. However, it seems that these factors are not likely to be the cause of increased iron absorption [11]. Another proposed mechanism for a probiotic (*L. fermentum*) effect on iron absorption is the release of an enzyme with ferric-reducing activity [19], although this mechanism seems unlikely, considering the observed effects in the present study, unless it is a species-specific effect ascribed to *L. fermentum*. There are also murine studies of probiotic effects on iron absorption [20]. However, it may be doubtful if these can be translated into the human situation because of the impact of intracellular ascorbic acid, which is a requirement for the function and regulation of DCYTB [21], and the fact that mice and rats produce ascorbic acid endogenously while humans do not may question their use in human iron absorption/metabolism studies. In addition, mice do not require DCYTB for absorption of iron from the diet [22].

## Figures and Tables

**Table 1:** Composition of the study products (per 200 mL drink and per capsule).

Composition	Oat Drink Lp299v	Oat Drink Control	MangoLp299v	MangoControl	Capsule Lp299v	Capsule Control
*L. plantarum* 299v (CFU) ^a^	7 × 10^8^	nd	1 × 10^9^	nd	1 × 10^10^	nd
Iron (mg) ^b^	4.1	4.0	4.0	4.1	4.2	4.2
Ascorbic acid (mg) ^c^	12.1	15.8	7.5	7.8	12	12
Folic acid (µg)	-	-	-	-	30	30

^a^*Lactobacillus plantarum* 299v; CFU: colony forming units. The content in the drinks was analysed by Probi AB with a modified NKML 140-2 method (2007); nd < 10 CFU/mL. ^b^ Ferrous lactate dihydrate in oat and mango drinks (200 mL) and ferrous fumarate in the capsules. Analysed by Eurofins Food & Agro Testing, Sweden (NKML method no 1391991). ^c^ Analysed by Eurofins Food & Agro Testing Sweden (Cereal Chemistry method). nd = not detectable.
